# Long-Term Functional Outcome After Early vs. Late Stoma Closure in Rectal Cancer Surgery: Sub-analysis of the Multicenter FORCE Trial

**DOI:** 10.1007/s12029-024-01062-2

**Published:** 2024-06-26

**Authors:** V. M. Meyer, N. Bosch, J. A. G. van der Heijden, A. J. Kalkdijk-Dijkstra, J. P. E. N. Pierie, G. L. Beets, P. M. A. Broens, B. R. Klarenbeek, H. L. van Westreenen

**Affiliations:** 1 Dept of Surgery, Isala Hospitals, Dokter Van Heesweg 2, 8025 AB Zwolle, The Netherlands; 2 Dept of Surgery, University Medical Center Groningen, PO Box 30.001, 9700 RB Groningen, The Netherlands; 3 Dept of Surgery, Radboud University Medical Center, Geert Grooteplein Zuid 10, 6525GA Nijmegen, The Netherlands; 4 Post Graduate School of Medicine (PGSOM), University Medical Center Groningen and University of Groningen, PO Box 30.001, 9700 RB Groningen, The Netherlands; 5 Dept of Surgery, Medical Center Leeuwarden, Henri Dunantweg 2, 8934 AD Leeuwarden, The Netherlands; 6 Department of Surgery, Netherlands Cancer Institute, Amsterdam, The Netherlands; 7 GROW School for Oncology and Developmental Biology, Maastricht University, Maastricht, The Netherlands

**Keywords:** Rectal cancer, Stoma closure, Low anterior resection, Quality of life, Anorectal function

## Abstract

**Purpose:**

The aim of this study was to assess the effect of early stoma closure on bowel function after low anterior resection (LAR) for rectal cancer.

**Methods:**

Patients participating in the FORCE trial who underwent LAR with protective stoma were included in this study. Patients were subdivided into an early closure group (< 3 months) and late closure group (> 3 months). Endpoints of this study were the Wexner Incontinence, low anterior resection syndrome (LARS), EORTC QLQ-CR29, and fecal incontinence quality of life (FIQL) scores at 1 year.

**Results:**

Between 2017 and 2020, 38 patients had received a diverting stoma after LAR for rectal cancer and could be included. There was no significant difference in LARS (31 vs. 30, *p* = 0.63) and Wexner score (6.2 vs. 5.8, *p* = 0.77) between the early and late closure groups. Time to stoma closure in days was not a predictor for LARS (*R*^*2*^ = 0.001, *F* (1,36) = 0.049, *p* = 0.83) or Wexner score (*R*^*2*^ = 0.008, *F* (1,36) = 0.287, *p* = 0.60) after restored continuity. There was no significant difference between any of the FIQL domains of lifestyle, coping, depression, and embarrassment. In the EORTC QLQ-29, body image scored higher in the late closure group (21.3 vs. 1.6, *p* = 0.004).

**Conclusion:**

Timing of stoma closure does not appear to affect long-term bowel function and quality of life, except for body image. To improve functional outcome, attention should be focused on other contributing factors.

## Introduction

TME surgery is the gold standard for resection of rectal carcinoma, leading to significant improvement in survival since its introduction by Heald [1]. Sphincter preserving techniques are preferred to avoid permanent stoma creation, something that is highly valued by patients [2]. However, the majority of patients suffer from impaired bowel function after low anterior resection (LAR) [3, 4]. As a consequence of improved cancer treatment, functional outcome is very important for long-term rectal cancer survivors [5].

Anastomotic leakage is a major complication after LAR and therefore a protective stoma to divert the flow of the feces externally is commonly used to minimize the possibility of an anastomotic leakage. Although a protective stoma will lead to a reduced rate of re-operation in case of a leakage, stoma-related morbidity such as dermatitis, high output, and/or herniation (occurring in up to 35% of patients) needs to be taken into account [6–8]. Also, protective stoma after LAR can contribute to a pathological microbiome, atrophy of the bowel wall musculature, and impaired mucosal absorptive function distally which all could affect bowel function after stoma closure [9–13]. Thus, early closure of a stoma could be beneficial. However, the heterogeneous literature on the subject does not allow for a clear consensus on optimal timing of stoma closure after LAR [12–14]. For example, most cohort studies do not report specifically on timing of stoma closure and randomized trials reporting on timing of closure are not powered for this outcome. Also, the decision to create a stoma is often left to the surgeon (i.e., more difficult cases) and often the effect of patient-related factors is not reported [15, 16].

Furthermore, a potential difference in functional outcome is difficult to investigate because several scoring systems are used to evaluate bowel function. Most frequently used after LAR is the LAR score, which is limited because it does not incorporate QoL or differentiate between incontinence and obstipation-related symptoms [17, 18]. In an attempt to capture the full extent of bowel-related problems, other frequently used validated bowel function and health-related quality of life (HRQoL) scores are being used such as the Fecal Incontinence Quality of Life (FIQL) scale, Wexner incontinence score, and the EORTC QLQ CR-29 questionnaire. These questionnaires are designed for different populations, answer different questions, and possess different validated psychometric properties while being used interchangeable, making comparison of studies difficult.

The aim of this study is to assess the effect of early stoma closure compared to late stoma closure, on bowel function and quality of life after LAR for rectal cancer.

## Methods

The FORCE trial was designed as a multicenter two-armed randomized controlled trial comparing the effect of pelvic floor rehabilitation (PFR) on functional outcome after rectal resection [19]. Patients participating in the FORCE trial who underwent LAR with temporary protective stoma were included in this study. Endpoints of this study were Wexner, LARS, EORTC QLQ-CR29, and FIQL scores at 1 year. For this study, patients were divided into two groups based on the timing of stoma closure. In the Netherlands, closure of a temporary stoma after uncomplicated rectal resection is generally planned 8–12 weeks after LAR. Patients who underwent stoma closure within 3 months were defined as the “early” closure group. Patients in the “late” closure group had their stoma closed after 3 months. Furthermore, a sub-analysis of “very late” closure group after 6 months and pelvic floor rehabilitation group was performed. The FORCE trial was approved by the Ethics Committee in Arnhem/Nijmegen, the Netherlands (reference number NL59799.091.16).

### Inclusion Criteria

Eligible patients underwent LAR for rectal cancer and were 18 years or older. Those with comorbidities such as inflammatory bowel disease or proctitis, a short life expectancy (< 1 year), locally advanced tumors that required extensive resections and those who had participated in biofeedback therapy in the last 6 months before the LAR procedure were excluded.

### Patients

This study was conducted in 2 academic and 15 teaching hospitals in the Netherlands between October 2017 and March 2020. Patients were asked to fill in the questionnaires 1 year after stoma closure. Demographic details, tumor characteristics, use of neo-adjuvant treatment, perioperative records including complications, and relevant history were registered prospectively. All patients provided written and verbal informed consent.

### Questionnaires

Functional outcome was measured through the DeFec questionnaire which contains four validated questionnaires: the LARS and Wexner incontinence score for bowel function and HRQoL through the FIQL and EORTC QLQ-CR29 questionnaires [20]. A multimodality approach was chosen; patients could fill in their questionnaires online or via mail. Patients were solicited through telephone calls in case of non-response.

The validated Wexner incontinence score ranges from 0 to 20. Wexner scores ≥ 1 were considered to be symptomatic (1–4: mild incontinence, 5–8: moderate incontinence, 9–20: severe incontinence). A clinically relevant difference was defined as minimally two points [21].

The validated Fecal Incontinence related Quality of Life score is composed of a total of 29 items; these items form four scales: lifestyle (10 items), coping/behavior (9 items), depression/self-perception (7 items), and embarrassment (3 items). A FIQL score of 1–4 represents poor to good QoL. A value of 0.4 was considered the minimal important change (MIC) for the FIQL in our sample [22].

The frequently validated LARS score consists of 5 subscales which amount to a score of 0–42 points. LARS score is divided into clinically significant subgroups of no LARS (0–20), minor LARS (21–29), and major LARS (30–42) [23].

The validated EORTC QLQ-CR29 is a tumor-specific HRQoL questionnaire for colorectal cancer patients. It consists of four scales and 19 individual items in Dutch and has been validated in neo-adjuvant-treated rectal cancer patients [24]. The diverse function and symptom scales range from 0 to 100, of which higher function scores resemble a better outcome and where a higher symptom score represents more complaints.

### Statistical Analysis

Descriptive statistics were obtained to identify any outliers and determine the distribution of data. If the assumptions for parametric testing were violated, a non-parametric alternative was used. Mean change in continuous data scores was compared using an analysis of variance (ANOVA). For categorical data, Chi-square or Mann–Whitney *U* test was used. Fisher’s exact test was used in case of small numbers (< 5). Categorical ordered data (such as the Wexner and LARS scores) were compared using the Jonckheere–Terpstra test for ordered alternatives. A linear regression model with time to stoma closure was fitted to predict bowel function (LARS and Wexner score). Time to stoma closure was defined as the number of days between index surgery and stoma closure. Analysis with correction of possible confounding factors was performed using ANCOVA. All variables that were possibly confounding (*p* < 0.2) between the early and late closure groups were included in multivariable analysis. Data were statistically significant at *p* < 0.05. All questionnaires were handled according to their manuals. IBM SPSS 23 was used [25].

## Results

### Patient-Related Outcomes

**Fig. 1:**
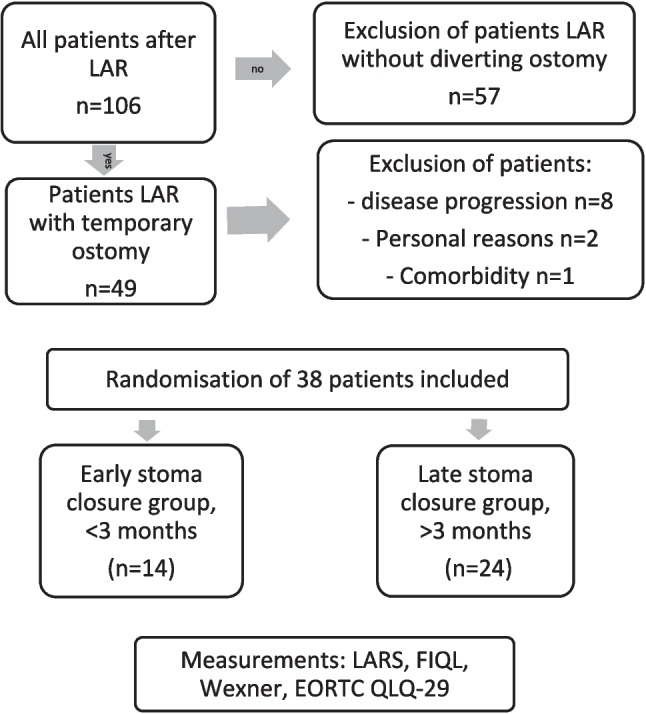
PRISMA flowchart of patient inclusion

Between October 2017 and March 2020, 106 patients were included in the FORCE trial. Fifty-seven patients underwent LAR without stoma, and the remaining 49 patients were eligible for this study. Forty-nine patients had received a protective stoma of which eight patients developed progression of disease, two patients withdrew due to personal circumstances, and one patient withdrew due to non-oncological co-morbidity and were therefore excluded. Finally, 38 patients with a protective stoma returning full questionnaires at 1-year follow-up were included (Fig. 1). Response rate of participating patients, measured after inclusion and randomization, was 91%.

There were no differences in age, sex, BMI, ASA score, cTNM classification, distance from anal verge, use of neo-adjuvant therapy, pelvic floor rehabilitation, length of stay, complications, or comorbidities for both groups (Table 1). Four patients had received their stoma later than index surgery due to complications.

**Table 1:** Patient-related and peri-operative factors*. p* in italic if > 0.05 < 0.10. SCRT, short-course radiotherapy. CRTx, chemoradiotherapy. *Median reported for “time to stoma closure” due to no Gaussian distribution

		**Stoma closure**				
		< 3 months 14 patients		> 3 months 24 patients		
		Mean^*^	Count	Mean*	Count	*p* value
**Age**		60		60		0.93
**Gender**	Male		11		15	0.47
	Female		3		9	
**BMI**		27.3		26.5		0.61
**ASA classification**	ASA 1		4		8	0.76
	ASA 2		7		13	
	ASA 3		3		3	
**Distance to anal verge (cm)**		6.7		6.4		0.71
**TNM cT-stadia**	cT1		1		1	0.80
	cT2		3		7	
	cT3		10		15	
**TNM cN-stadia**	cN0		7		8	0.40
	cN1		3		10	
	cN2		4		5	
**TNM cM-stadia**	cM0		14		19	0.26
	cM1		0		3	
	cMx		0		1	
**Neo-adjuvant therapy**	yes		10		17	0.94
	no		4		7	
**Type of surgery**	Laparoscopic		12		14	0.19
	Robot		2		8	
	Conversion		0		2	
**Pelvic floor rehabilitation**	Yes		7		10	0.38
	No		7		14	
**Time to closure in days (median)**		67		139		
**Length of stay in days**		7		10		0.24
**Blood loss during surgery in cc**		34		67		0.26
**Surgical reintervention**	Yes		0		6	*0.07*
	No		14		17	
**Radiological intervention**	Yes		0		1	1.0
	No		14		22	

### Bowel Function and Health-Related Quality of Life

There was no significant difference in LARS (31 vs. 30, *p* = 0.63) and Wexner score (6.2 vs. 5.8, *p* = 0.77) between the early and late closure groups. The prevalence of major LARS and categorical Wexner scores was not statistically different between groups (Table 2). Linear regression analysis did not reveal time to stoma closure as predictors for LARS (*R*^*2*^ = 0.001, *F* (1,36) = 0.049, *p* = 0.83) or Wexner score (*R*^*2*^ = 0.008, *F* (1,36) = 0.287, *p* = 0.60) after restored continuity.

**Table 2:** Functional outcome parameters and 1 year after stoma closure. Cat, categorical

		**Functional outcome 1 year after stoma closure**				
		< 3 months 14 patients		> 3 months 24 patients		
		Mean	Count	Mean	Count	*p* value
**LARS score**		31		30		0.63
**LARS cat**	No or minor		4		12	0.31
	Major		10		12	
**FIQoL**	Lifestyle	2.51		2.78		0.46
	Coping	2.18		2.33		0.68
	Depression	2.56		2.66		0.79
	Embarrassment	2.48		2.46		0.97
**Wexner**		6		6		0.76
**Wexner cat**	No symptoms		0		2	0.60
	Mild		7		8	
	Moderate		3		8	
	Severe		4		6	

**Fig. 2:**
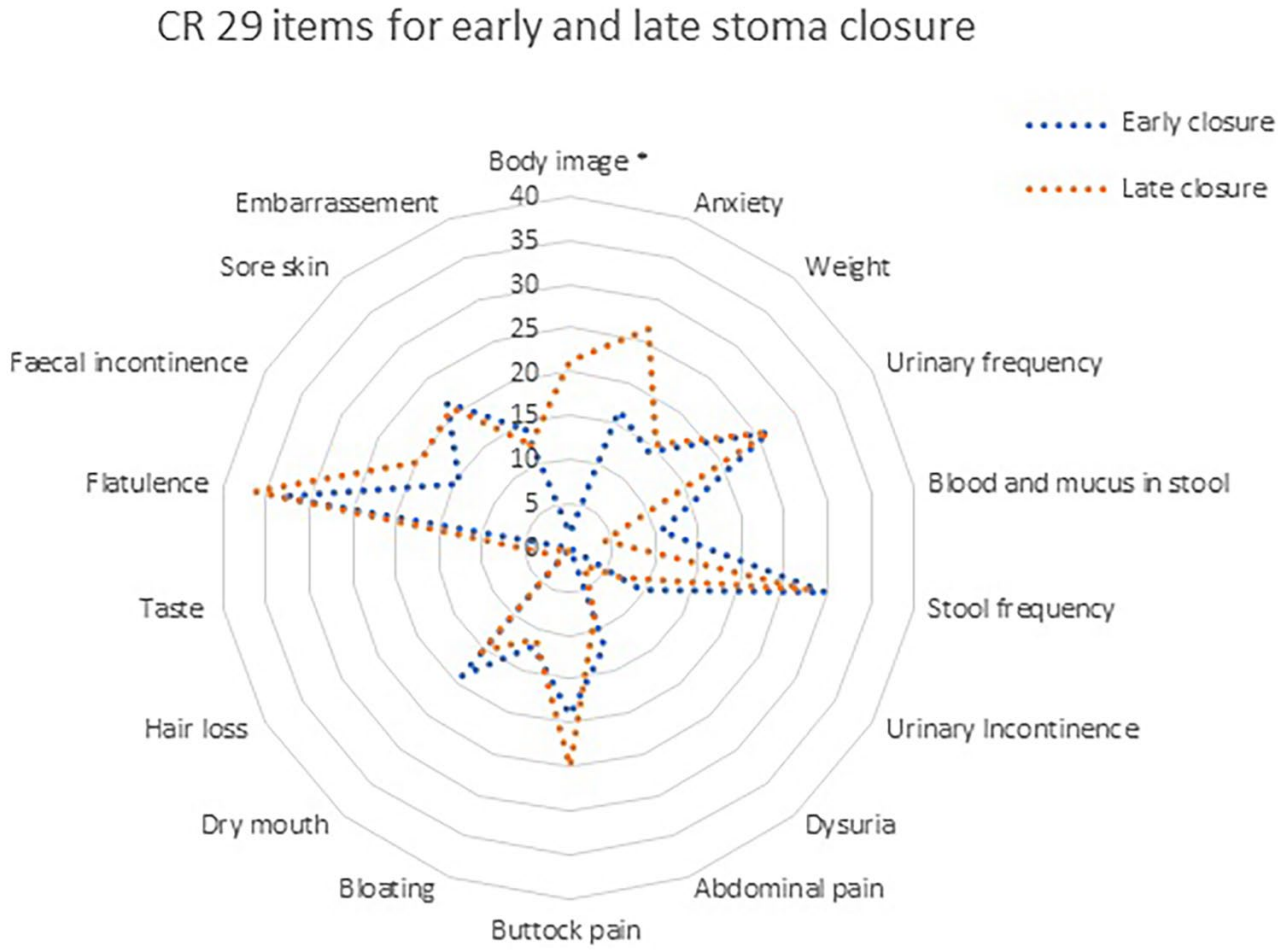
CR 29 item scores for early closure (< 3 months) and late closure (> 3 months) groups at 1 year. * denotes significance at *p* < 0.05

There was no significant difference between any of the FIQL domains of lifestyle, coping, depression, and embarrassment (Table 2). In the EORTC QLQ-29, body image scored higher in the late closure group (21.3 vs. 1.6, *p* = 0.004) (Fig. 2). Body image in the late closure group remained significantly higher in multivariable analysis after correction for anastomotic leak, operating time, complications after surgery, length of stay, type of surgery, and M1 disease (*p* = 0.02). There was no significant difference between the other items of the QLQ-29 (Fig. 2).

Sub-analysis of groups with and without PFR both did not show a significant difference between early and late closure for mean Wexner score (*p* = 0.49 and 0.97), LARS score (*p* = 0.36 and 0.59), or any of the FIQL and CR-29 domains. For the group of patients with very late closure (defined as > 6 months), we found no significant differences for LARS (*p* = 0.58), Wexner (*p* = 0.28), or any of the Qol (FIQL and CR-29) domains (*p* > 0.4) in 10 patients. Three of these patients received PFR. Patients undergoing stoma closure after 6 months had significantly more anastomotic leaks (*p* < 0.001), admission days (*p* < 0.001), and a trend towards more postoperative complications at index surgery (*p* = 0.08). There was no significant difference in LARS (*p* = 0.73), Wexner (*p* = 0.53), or any of the FIQL domains (*p* > 0.75) between 7 patients requiring a reintervention and 31 patients who did not. No patients died.

## Discussion

Stoma closure within 3 months does not appear to improve long-term bowel function or HRQoL, as measured by the Wexner, LARS, QLQ CR-29, and FIQL scores.

In this study, we did not find a significant correlation between time to stoma closure and LARS or Wexner score. Although this has been reported, literature on this subject is scarce hindering a proper comparison of studies [15, 26]. The latest review on the subject by Podda et al. including 7 RCT’s could not find a difference in LARS between early (< 30 days) vs. late (> 60 days) stoma closure [27]. Vogel et al. performed an extensive pooled analysis of 719 patients including 4 RCT’s comparing early versus late closure and found a mean difference in closure time of 2.39 months between no and major LARS groups (95% CI, 1.28–3.51, *p* < 0.0001: *I*2 = 21%, *X*^2^ = 0.28) [28]. However, median time to closure varied from 2.4 to 15.6 months. In their comprehensive review, a proposition for timing of stoma closure could not be provided. They also reported that 5 out of 6 included studies did not find a significant association between LARS and timing of closure [28].

Observing the high variability in interval to stoma closure, a sub-analysis for interval > 6 months was performed in which we found no significant differences for LARS, Wexner, FIQL, and CR-29 scores in 10 patients. Hughes et al. showed that stoma closure within 6 months is protective for major LARS (OR 0.2, 95% CI, 0.1–0.3, *p* < 0.01), and after 1 year it becomes associated with major LARS (OR 3.7, CI 95%, 1.1–13.1, *p* = 0.03). Obviously, such late closure of a diverting ostomy is often related to a complicated clinical course which could influence functional outcome [29, 30]. More anastomotic leaks, admission days, and a trend towards more postoperative complications were found in patients who underwent stoma closure > 6 months. Although a small sample size prohibited a formal analysis, worse functional outcome after “very” late closure could very well represent an anastomosis-related complication rather than an effect of timing of stoma closure.

Although no difference in bowel function was found between early and late groups, body image was significantly better in the late closure group. This result appears to be in line with a secondary analysis of the EASY study that examined health-related quality of life (HRQOL) following early versus late closure of a temporary ileostomy. This study also showed improved QoL parameters (less bodily pain with increased mental health at 12 months, *p* < 0.05 for both) for their late closure group [31]. It has been shown that patients dealing with chronic conditions and cancer appear to reset internal values and even report higher QoL than their healthy peers. This so-called “response shift” illustrates a change in perspective on life and is common in colorectal cancer survivors [4, 32]. Thus, the observed improved QoL properties in patients undergoing late closure (often due to complications) could be explained by a more pronounced response shift in this particular group of patients.

A limitation is the sample size of this study, prohibiting more extensive analysis. Like most studies reporting on timing of stoma closure, the FORCE trial was not powered for this outcome making our study theoretically more susceptible to falsely accepting that timing of stoma closure does not affect outcome (type 2 error). Also, the study protocol did not include data on morbidity of stoma closure, which is relevant in the discussion of timing of stoma closure and should therefore be included in future analyses. The decision to create a stoma was a pragmatic approach of the surgeon ensuring optimal treatment for the individual patient but could introduce selection bias. In the late closure group, there was a trend towards more anastomotic leakage, which could have impacted functional outcomes.

There are many factors that impact on functional outcome. Coping mechanisms, response shift and low anastomoses, radiotherapy and anastomotic leakage will influence the perceived bowel function [14, 32–34]. Timing of stoma closure could be a contributing factor but is probably not a highly important one. Other factors such as dose adjustment and more fractioning of radiotherapy have shown to improve functional outcome [35–37]. Furthermore, organ-sparing treatment (when possible) will lead to better functional results than resection [38]. Also, there are indications that bowel dysfunction after stoma closure could be temporary [18, 28]. For example, Gadan et al. found in a 12-year follow-up of their RCT comparing anorectal function after protective stoma that there was no difference in categorical LARS incidence, but specific symptoms did occur more often in temporary ostomates [10]. And finally, the stoma itself appears to be a more important factor than timing of closure. Vogel et al. showed in their review of 7 studies that major LARS occurred 2.84 times more often in patients with a stoma. Up to 9% of patients develop a serious complication following stoma closure requiring re-operation or ICU, one in five is readmitted within 30 days of stoma creation, and, often underreported, up to 35% of patients develop an incisional hernia after stoma reversal of which two-thirds require a re-operation [8, 39, 40]. This had led to a change in strategy towards highly selective use of protective stoma combined with pro-active leakage management in certain centers that now report a high bowel continuity rate and lower readmission rates without increased leakage, re-operation, or mortality [41–43]. This suggests that a standard diverting ostomy is perhaps not the risk-adverse strategy we once thought but maybe should be reserved for a selected group of patients [41]. The GRECCAR-17 trial, comparing quality of life between selective vs. standard use of diverting ostomy after LAR for cancer, is now underway [44]. Overall, attention should be focused on other contributing factors then timing of stoma closure to improve functional outcome after LAR for rectal cancer.

## Conclusion

Timing of stoma closure does not appear as an important factor in long-term bowel function and HRQoL. To improve functional outcome, attention should be focused on other contributing factors.

## Data Availability

The datasets generated during and/or analyzed during the current study are available from the corresponding author on reasonable request.
